# Editorial: Hereditary Periodic Fevers and Autoinflammatory Diseases

**DOI:** 10.3389/fped.2022.855738

**Published:** 2022-02-14

**Authors:** Hafize Emine Sönmez, Betül Sözeri, Nuray Aktay Ayaz

**Affiliations:** ^1^Department of Pediatric Rheumatology, Kocaeli University, Izmit, Turkey; ^2^Department of Pediatric Rheumatology, Ümraniye Research and Training Hospital, University of Health Sciences, Istanbul, Turkey; ^3^Department of Pediatric Rheumatology, School of Medicine, Istanbul University, Istanbul, Turkey

**Keywords:** autoinflammatory diseases, familial Mediterranean fever, PFAPA, colchicine, rheumatology

Systemic autoinflammatory diseases (SAIDs) are characterized by unprovoked exaggerated inflammation without significant levels of autoantibodies or antigen-specific T cells. Hereditary periodic fevers (HPFs) are considered the prototype of SAIDs and are defined as three or more unexplained episodes of fever occurring at least 7 days apart in a 6-month period (1). With the advance of molecular studies, it is well-known now that SAIDs are growing family of disorders including many diseases in which fever is not predominant. Since the SAIDs occupy a substantial place in pediatric rheumatology practice, the Research Topic focused on the clinical aspects, and treatment approaches in this area appears to be critical.

Out of eight manuscripts submitted to the journal six were accepted (2). Two articles focused on the clinical findings, one on the biomarkers, and the remaining three on the treatment approaches. The first article penned by Öztürk et al. evaluated a large cohort of pediatric patients with familial Mediterranean fever (FMF) and reported the allele frequencies of common mutations as follows: M694V (55.3%), M680I (11.3%), V726A (7.6%), and E148Q (7.2%). This article displays the largest pediatric cohort to date presenting the phenotype-genotype correlation in FMF. In another study, Veres et al. discussed the effect of a positive family history on the periodic fever, aphthous stomatitis, pharyngitis, and adenitis (PFAPA) phenotype. They concluded that PFAPA patients with a positive family history expressed a different subset of disease with a higher frequency of arthralgia, myalgia, carrying M694V mutation, and better response to colchicine compared to PFAPA patients without a positive family history. The study examining the biomarkers by Omma et al. suggested that serum endocan levels may be a potential biomarker to distinguish colchicine-resistant FMF patients. They showed that colchicine-resistant FMF patients had higher serum endocan levels compared to colchicine-responsive FMF patients. Unlike other acute phase reactants, endocan levels were high with or without an attack in colchicine resistant FMF patients. The remaining three articles were about treatment approaches in SAIDs. Welzel et al. evaluated the effectiveness of colchicine in PFAPA patients. Their study includes 27 patients with PFAPA of whom 14 (52%) had no flare under the colchicine treatment while the frequency of flares improved with colchicine in 24 (88.8%) patients. Consequently, the authors introduced colchicine as a well-tolerated and safe drug acting via decreasing disease activity, and frequency and shortening duration of flares. Yücel et al. evaluated the effectiveness of canakinumab in 65 patients with FMF. Canakinumab was prescribed subcutaneously every 4 weeks for 12 months, when the complete remission was achieved dose interval was extended to every 1.5 months for 6 months, then every 2 months for 6 months, then every 3 months for a year, and finally if there was no disease activation, canakinumab treatment was ceased at the end of 3 years. Complete remission was present in 57 (87.6%) and partial remission in seven (10.7%) patients. Fingerhutová et al. reported their clinical experiences with anakinra treatment in patients with SAIDs. They reviewed the clinical records of 47 patients and presented that anakinra was a well-tolerated and safe drug despite in case of using higher than recommended doses.

Although SAIDs are rare diseases, we hope this collaborative special Research Topic may provide guide to physicians who encounter these diseases in clinical practice.

## Author Contributions

All authors listed have made a substantial, direct, and intellectual contribution to the work and approved it for publication.

## Conflict of Interest

The authors declare that the research was conducted in the absence of any commercial or financial relationships that could be construed as a potential conflict of interest.

## Publisher's Note

All claims expressed in this article are solely those of the authors and do not necessarily represent those of their affiliated organizations, or those of the publisher, the editors and the reviewers. Any product that may be evaluated in this article, or claim that may be made by its manufacturer, is not guaranteed or endorsed by the publisher.
